# A Deserving Honor

**Published:** 1887-01

**Authors:** 


A Deserving Honor.—It is with pleasure we announce that Dr. Judson B. Andrews, Superintendent of the Buffalo State Asylum, has been selected as president of the section on psychology and nervous diseases of the International Medical Congress.

Dr. Andrews is well-known, both at home and abroad, by his articles which he has contributed to medical science during the last twenty years. Dr. John P. Gray had been elected as the person well calculated to fill the position. By his death his mantle worthily falls on Dr. Andrews as the one best fitted by education and natural acquirements to fill such a position. The work of this section has already favorably progressed. Several English neurologists have signified their intention of being present and reading papers.

---

WE call attention to the advertisement of Messrs. Wyeth & Bro., of new combinations of patent drugs which they offer in the form of Hypodermic Tablets. A long experience with the preparations of this firm has caused us to look upon the name of "Wyeth" as a guarantee of purity and excellence. Our readers will find in the list many new remedies, and these preparations make their employment very convenient. In their full list is now comprised every known remedy suitable for hypodermic exhibition.

---

THE STATE MEDICAL SOCIETY will hold its regular annual meeting in Albany, Feb. 2d, 3d and 4th. The programme arranged is most elaborate. It will doubtless prove a source of pleasure to all who attend.

